# Fatty acids and risk of dilated cardiomyopathy: A two-sample Mendelian randomization study

**DOI:** 10.3389/fnut.2023.1068050

**Published:** 2023-02-16

**Authors:** Jiexin Zhang, Qiang Luo, Jun Hou, Wenjing Xiao, Pan Long, Yonghe Hu, Xin Chen, Han Wang

**Affiliations:** ^1^Department of Laboratory Medicine, Affiliated Hospital of Southwest Jiaotong University, The Third People's Hospital of Chengdu, Chengdu, Sichuan, China; ^2^Central Laboratory, The General Hospital of Western Theater Command, Chengdu, Sichuan, China; ^3^Department of Cardiology, Affiliated Hospital of Southwest Jiaotong University, The Third People's Hospital of Chengdu, Chengdu, Sichuan, China

**Keywords:** oleic acid, hydroxy fatty acid, dilated cardiomyopathy, two-sample Mendelian randomization, genome-wide association studies

## Abstract

**Background:**

Previous observational studies have shown intimate associations between fatty acids (FAs) and dilated cardiomyopathy (DCM). However, due to the confounding factors and reverse causal association found in observational epidemiological studies, the etiological explanation is not credible.

**Objective:**

To exclude possible confounding factors and reverse causal associations found in observational epidemiological studies, we used the two-sample Mendelian randomization (MR) analysis to verify the causal relationship between FAs and DCM risk.

**Method:**

All data of 54 FAs were downloaded from the genome-wide association studies (GWAS) catalog, and the summary statistics of DCM were extracted from the HF Molecular Epidemiology for Therapeutic Targets Consortium GWAS. Two-sample MR analysis was conducted to evaluate the causal effect of FAs on DCM risk through several analytical methods, including MR-Egger, inverse variance weighting (IVW), maximum likelihood, weighted median estimator (WME), and the MR pleiotropy residual sum and outlier test (MRPRESSO). Directionality tests using MR-Steiger to assess the possibility of reverse causation.

**Results:**

Our analysis identified two FAs, oleic acid and fatty acid (18:1)-OH, that may have a significant causal effect on DCM. MR analyses indicated that oleic acid was suggestively associated with a heightened risk of DCM (OR = 1.291, 95%CI: 1.044–1.595, *P* = 0.018). As a probable metabolite of oleic acid, fatty acid (18:1)-OH has a suggestive association with a lower risk of DCM (OR = 0.402, 95%CI: 0.167–0.966, *P* = 0.041). The results of the directionality test suggested that there was no reverse causality between exposure and outcome (*P* < 0.001). In contrast, the other 52 available FAs were discovered to have no significant causal relationships with DCM (*P* > 0.05).

**Conclusion:**

Our findings propose that oleic acid and fatty acid (18:1)-OH may have causal relationships with DCM, indicating that the risk of DCM from oleic acid may be decreased by encouraging the conversion of oleic acid to fatty acid (18:1)-OH.

## 1. Introduction

Dilated cardiomyopathy (DCM) is a type of heart muscle disease that causes left ventricular dilation and systolic dysfunction (1). This abnormal structure or function of the myocardium can develop into complications such as sudden cardiac death and intractable heart failure, and can therefore ultimately be life-threatening (2). Furthermore, chronically treated patients may occasionally exhibit acute decompensated heart failure (2). Globally, DCM is the most frequent cardiomyopathy, occurring in people of any age or gender (2). As early as 2013, the prevalence of DCM increased from 1/2,500 to 1/250 (3). DCM is classified as a serious heart disease by the World Health Organization because of its high morbidity and mortality rates (4). Due to its high hospital admission rates and the potential need for a heart transplant, DCM imposes significant financial obligations (2). As a consequence, despite advances in DCM treatment, outcomes still need to be improved. A significant advancement in DCM clinical therapy will be made if an intervention that effectively prevents or treats DCM without imposing a heavy financial burden on patients and with excellent compliance is Id.

DCM is a sophisticated form of ventricular remodeling that alters the morphology of the ventricles and causes myocardial fibrosis. In this condition, all chambers of the heart enlarge, ventricular wall pressure rises, and systolic function decreases (2). DCM is characterized by an enlarged and weakened heart muscle, and patients often present with weakness, dizziness, pallor, dyspnoea, edema, abdominal distention, and even embolism (5). Besides, patients with DCM are always accompanied by mitral regurgitation, ventricular arrhythmias, and other rhythm disturbances (2). An electrocardiogram, cardiac MRI features, history-taking, and clinical findings all play a role in the clinical diagnosis (6). Endocardial biopsy and the use of biomarkers like B-type natriuretic peptide can also assist with the diagnosis and confirmation of the disease process (7, 8). DCM has a complex multifactorial etiology that includes both inherited and environmental factors (2). The most important cause of dilated cardiomyopathy is genetic, with the main mode of inheritance being autosomal dominant (2). The pathogenic genes of DCM mainly encode cytoskeletal proteins and sarcomeric proteins. Non-genetic triggers of DCM include tachycardia-induced cardiomyopathy, hypertension, alcohol or cocaine abuse, inflammatory disorders, and autoimmune diseases (2). Evidence of inflammatory cell infiltration and gene expression patterns compatible with immune cell activation is often revealed by pathological examination of myocardial biopsy samples (or autopsies) of patients with DCM (4, 9). Abnormalities in myocardial fatty acid metabolism play an important role in DCM and can trigger a range of events such as impaired energetics, and oxidative stress and even lead to reduced cardiac function (10). Neglia et al. showed that patients with DCM have reduced levels of fatty acid oxidation (11). As DCM pathogenesis research advances, identifying its potential targets can serve as a foundation for new interventions.

Fatty acids (FAs) are essential nutrients for the human body. The diet-heart hypothesis is primarily based on the effect of fatty acids in dietary fat on blood lipids, which in turn modulate cardiovascular outcomes by affecting thrombosis, endothelial function, inflammation, arrhythmias, and the onset of diabetes (12). FAs are the primary metabolic substrates for myocardial action in the heart under aerobic conditions and have an impact on processes like ion channel function and cell signaling (13). Yazaki et al. first investigated the metabolic process of fatty acids using ^123^I-β-methyl-p-iodophenyl pentadecanoic acid (BMIPP) single photon emission computed tomography (SPECT) technology labeling in 1999 and discovered that the severity of abnormal fatty acid metabolism in DCM myocardia reflected the severity of hemodynamic deterioration and histological abnormalities (14). N-3 polyunsaturated fatty acids (PUFAs) seemed to reduce the risk of arrhythmia in patients with DCM, according to data from the Microvolt T-Wave Alternans (MTWA) test, which has been demonstrated to be one of the best predictors of severe arrhythmias and sudden death (15). According to the findings of DCM metabolomics studies, linoleic acid could be used as the primary biomarker to measure the effectiveness of DCM treatment, and hydroxyl fatty acid esters were potential biomarkers to identify DCM (16). Additionally, Nitro-oleic acid (NO_2_-OA), a signaling mediator produced naturally by reacting oleic acid with nitrogen dioxide (NO_2_), was created as a therapeutic agent for fibrotic and inflammatory diseases and has been proven effective in numerous animal models of cardiovascular diseases (17). Controversially, several *in vitro* and animal investigations have raised concerns that linoleic acid and other n-6 PUFAs may induce inflammation and thrombosis (18). Clinical trial results also suggest an increase in arrhythmia occurrences in patients with ventricular tachycardia who are treated with n-3 PUFAs (19). The heterogeneity between trials may be brought about by variations in the research populations and dietary sources of fatty acids. Therefore, it is difficult to be convinced that FAs have a causal effect on DCM and which FA regulation might be most important in DCM.

Previous observational studies have shown that FAs are substantially associated with DCM, but traditional observational studies are prone to confounding. For the confounding factors that can be observed in such studies, covariable correction methods can make comparisons between groups that tend to be balanced. However, for unmeasured confounders, even the best epidemiological design cannot correct for bias. Therefore, we introduced an instrumental variables (IVs) model to simulate sample randomization allocation in order to eliminate the bias effects of any confounding factors, other than target exposure, on causal inference (20). Mendelian randomization (MR), a research method proposed by Katan in 1986, infers and analyzes causal effects by establishing an instrumental variable model utilizing single-nucleotide polymorphisms (SNPs) (20). Genetic variation, according t' Mendel's law, is independent of environmental influences and avoids the influence of confounding factors. Simultaneously, genetic variation as a starting point of distinct outcomes might rule out the potential reverse causality possibility (21). Consequently, we may obtain the causal effect of “exposure outcome” using the MR approach, which cannot be determined using the usual observational research design. In our study, we employed a two-sample MR approach to analyze GWAS data to evaluate the causal relationship between FAs and DCM risk.

## 2. Methods

### 2.1. Study design

**Figure 1:**
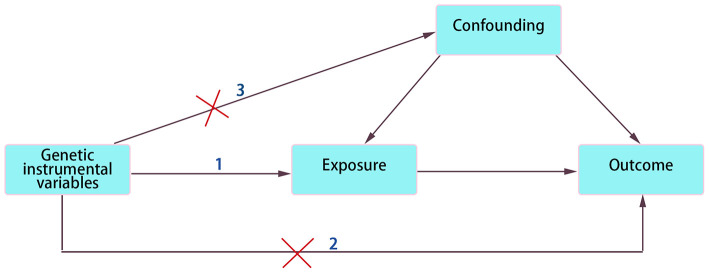
Schematic diagram of two-sample MR analysis. The three assumptions in MR model: 1. Relevance. IVs must be strongly related to exposure factor. 2. Exclusivity. IVs affect outcome only through exposure factor not through any other pathways. 3. Independence. IVs and confounding factors are independent of each other.

As shown in the schematic diagram in Figure 1, we employed the two-sample MR approach to confirm the causality between FAs and DCM. Each FA was designed as an exposure factor, while DCM served as an outcome indicator.

### 2.2. Data source

All data used are openly accessible. SNPs associated with different FAs were extracted from four major large clinical studies (Table 1). (a) Some genetic associations between SNPs and FAs were obtained from the genome-wide association study (GWAS) by Wu et al. (22), which consists of 5 cohorts, namely the Atherosclerosis Risk in Communities (ARIC) Study, the Cardiovascular Health Study (CHS), the Coronary Artery Risk Development in Young Adults (CARDIA) Study, the Invecchiare in Chianti (InCHIANTI) Study, and the Multi-Ethnic Study of Atherosclerosis (MESA). Among them, ARIC and CHS, which are from the Cohort for Heart and Aging Research in Genomic Epidemiology (CHARGE), were the prominent data contributors. Only 8,961 European participants from these cohorts were included in this GWAS data set. (b) The genetic loci of PUFAs were also obtained from the five above mentioned population-based cohorts for GWAS. Differently, Rozenn et al. selected 8,866 subjects of European descent for genetic analysis (23). (c). The LURIC Health Study, from 1997 to 2000, involved 3,316 German Caucasians (2,309 men and 1,007 women), and excluded participants who had had an acute illness (except acute coronary syndrome), non-cardiac chronic illness, or malignant neoplasms within the past 5 years (24). This prospective cohort study assessed genetic and environmental risk factors for cardiovascular disease in patients. Participants were filtered to remove individuals under the age of 18; persons with absent covariates including age, waist–hip ratio, BMI, and sex; and samples with a < 99% sample call rate. Finally, a total of 687,262 SNPs were obtained from 3,061 samples (25). (d) Other genetic data were acquired from 5,662 controls in the Pakistan Myocardial Infarction Risk Study (PROIS) and 13,814 UK participants in the INTERVAL study (26). PROIS is a case–control study of first-ever acute myocardial infarction in nine urban centers in Pakistan, and INTERVAL is a prospective cohort study of approximately 50,000 blood donors from the United Kingdom. These final samples were screened strictly with genetic and lipid characteristics. Blood samples were collected for measurement and analysis to obtain levels of each fatty acid. The plasma phospholipids were separated by TLC, and the fatty acids were subsequently quantified by gas chromatography. Plasma total fatty acids in the INCHIANTI cohort were determined using a similar GC technique. Serum lipid levels were quantified using a high-resolution mass spectrometer. In our study, considering that gene loci of these fatty acids rarely reach genome-wide significance in GWAS, SNPs with suggestive genome-wide significance thresholds (i.e., *P* < 5 × 10^−5^) were selected as IVs.

**Table 1:** Fatty acids and dilated cardiomyopathy summary data sources.

**Trait**	**Year**	**Study**	**Sample**	**Subjects**	**Case**	**Control**
Palmitic acid palmitoleic acid stearic acid oleic acid	2013	ARIC	Plasma phospholipid	3,269	NA	NA
	2013	CARDIA	Plasma phospholipid	1,507	NA	NA
	2013	CHS	Plasma phospholipid	2,404	NA	NA
	2013	InCHIANTI	Total plasma	1,075	NA	NA
	2013	MESA	Plasma phospholipid	706	NA	NA
Alphalinolenic acid docosahexaenoic acid docosapentaenoic acid eicosapentaenoic acid	2011	ARIC	Plasma phospholipid	3,268	NA	NA
	2011	CARDIA	Plasma phospholipid	1,507	NA	NA
	2011	CHS	Plasma phospholipid	2,326	NA	NA
	2011	InCHIANTI	Total plasma	1,075	NA	NA
	2011	MESA	Plasma phospholipid	690	NA	NA
Arachidonic acid dihomo-gamma-linolenic acid linoleic acid linolenic acid myristic acid trans-palmitoleic acid 9-cis,12-trans octadecanoic acid 9-trans,12-cis octadecanoic acid 9-trans,12-trans octadecanoic acid	2020	LURIC	Serum	3,061	NA	NA
Fatty acid (22:0) Fatty acid (24:0)	2015	CHARGE	Plasma phospholipid	10,129	NA	NA
Fatty acids^1^	2021	INTERVAL	Serum	13,814	NA	NA
	2009	PROMIS	Serum	5,662	NA	NA
Dilated cardiomyopathy	2021	HF METTC	Serum	533,543	1,861	531,682

^1^ Including: Fatty acid (12:0), Fatty acid (12:1), Fatty acid (14:0), Fatty acid (14:1), Fatty acid (15:0), Fatty acid (16:0), Fatty acid (16:0)-OH, Fatty acid (16:1), Fatty acid (16:1)-OH, Fatty acid (16:2), Fatty acid (16:3), Fatty acid (17:0), Fatty acid (17:1), Fatty acid (18:0), Fatty acid (18:0)-OH, Fatty acid (18:1), Fatty acid (18:1)-OH, Fatty acid (18:2), Fatty acid (18:2)-OH, Fatty acid (18:3), Fatty acid (18:4), Fatty acid (19:0), Fatty acid (19:1), Fatty acid (20:1), Fatty acid (20:2), Fatty acid (20:3), Fatty acid (20:4), Fatty acid (20:5), Fatty acid (21:0), Fatty acid (22:0), Fatty acid (22:3), Fatty acid (22:4), Fatty acid (22:5), Fatty acid (22:6), Fatty acid (23:0), Fatty acid (24:0), Fatty acid (24:1).

ARIC, Atherosclerosis Risk in Communities; CARDIA, Coronary Artery Risk Development in Young Adults; CHARGE, Cohort for Heart and Aging Research in Genomic Epidemiology; CHD, coronary heart disease; CHS, Cardiovascular Health Study; HF METTC, Heart Failure Molecular Epidemiology for Therapeutic Targets Consortium; InCHIANTI, Invecchiare in Chianti; LURIC, Ludwigshafen Risk and Cardiovascular Health; MESA, Multi-Ethnic Study of Atherosclerosis; PROIS, Pakistan Myocardial Infarction Risk Study.

Summary statistics for genetic IVs of DCM were extracted from the largest available GWAS meta-analysis with European ancestry performed by the Heart Failure (HF) Molecular Epidemiology for Therapeutic Targets Consortium (27). For the DCM GWAS, they included 533,543 European individuals (1,861 cases and 531,682 controls), and the age, gender and principal components of patients was adjusted.

### 2.3. Genetic instrumental variables

In MR analysis, IVs must satisfy three core characteristics: (1) Relevance. There needs to be a strong relationship between IVs and exposure factors; otherwise, causal effects will have bias. (2) Exclusivity. IVs can only be associated with outcome through exposure factors, not through any other pathways. (3) Independence. Random distribution of alleles provides a theoretical basis for the independence of IVs and confounding factors. Hence, all data for which a battery of steps was performed in controlling the quality of selected SNPs were eligible. To ensure a strong correlation between IVs and oleic acid, the filter criteria (*P* < 5 × 10^−8^, *r*^2^ < 0.001, genetic distance = 10,000 KB, minor allele frequency >0.01) were set using Plink Software. Importantly, the selected SNPs are not in linkage disequilibrium (LD) (28). Then, we searched the Catalog and PhenoScanner database to find and exclude SNPs which had relationships with known confounders, to meet the standard of the independence of genetic variation and confounders. Next, we calculated the F statistic, and abandoned SNPs with an *F*-value < 10 to avoid bias (29). Those satisfactory SNPs whose F values were significantly >10 after stringent screening were used for the following MR analysis.

### 2.4. Mendelian randomization analysis

The Mendelian randomization method defines genetic variation as IVs and solves the bias effect of confounding factors on causal judgment to a large extent by introducing the IVs model. In the current study, we utilized a two-sample MR design approach and combined different summary statistical methods to effectively infer the causal relationships between exposure factors and outcomes based on different MR hypotheses, including the inverse variance weighted (IVW) method, MR-Egger, the MR pleiotropy residual sum and outlier test (MRPRESSO), maximum likelihood and weighted median estimator (WME). In addition, we used MR-Steiger to verify whether there is a potential reverse causal possibility between FAs and DCM (30).

The IVW method was predominant in our MR analysis. It assumes that all bias is zero and produces a combined estimate of causal effects by using a combination of the Wald ratio and the selective fixed effects model, that is, the inverse variance weighted mean (IVW estimate) (31). The accuracy of this estimate is higher than that of any causal effect estimate based on a single genetic IV. The outliers in IVW estimates were then detected and corrected with MRPRESSO. Then, using MR-Egger, maximum likelihood, WME, and consistent estimates of causal effects, the relationships between exposure factors and outcome were further verified. MR-Egger is a weighted linear regression method based on the InSIDE hypothesis that can give valid tests and consistent estimates of causal effects even if all IVs are invalid (32). The WME estimate is more significant than the simple median, and it is calculated based on the weighted empirical distribution function for the ratio estimate of a single SNP (33). The odds ratio (OR) and 95%CI were calculated to explain the results of MR analysis. To interpret the multiple testing in this study, we used the Bonferroni correction for significance level. *P*-value between 9.26 × 10^−4^ (0.05 divided by 54 risk factors) and 0.05 were considered to be potentially associated. All analyses in our study were carried out using R (Version 4.1.2, https://www.r-project.org/) software package, two-Sample MR and MRPRESSO packages.

### 2.5. Heterogeneity and pleiotropic evaluates

To ensure the assumption of exclusivity, we performed heterogeneity and pleiotropy analyses on the included IVs. The intercept of MR-Egger regression is an important indicator of SNPs' potential pleiotropy. The closer the intercept value is to zero, the smaller the effect of genetic pleiotropy. Heterogeneity was analyzed mainly by MRPRESSO method and Cochran Q test. Meanwhile, we carried out “leave one-out” sensitivity analysis of the remaining SNPs by the IVW method after excluding one SNP, hoping to evaluate the effect of the individual SNP on DCM. If the *p*-value is not significant, it indicates that there is no bias and heterogeneity in the included IVs. On the contrary, if the *p*-value is < 0.05, bias and heterogeneity exist.

## 3. Results

**Figure 2:**
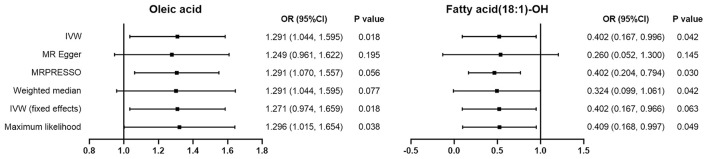
The odds ratio (OR) results for the causal effects with oleic acid and DCM, fatty acid (18:1)-OH and DCM respectively by using MR methods. The small black square represents the point estimate of the study effect size (OR). The length of the line segment represents the 95% confidence interval (95%CI) for each effect value for each study.

All 54 available FAs' data were extracted. The SNPs of each FA were strictly screened in turn, and the included SNPs were selected as IVs for multiple calculation and verification. After calculation, we found that two FAs, oleic acid and fatty acid (18:1)-OH, were suggestively associated with DCM (Figure 2). However, the other 52 FAs had no significant causal relationships with DCM risk (Supplementary material). The detailed MR analysis results of oleic acid and fatty acid (18:1)-OH are as follows.

### 3.1. Genetic instrumental variables filtered

#### 3.1.1. Oleic acid

SNPs with low allele frequencies ≤ 0.01 or no meaningful genome-wide association evidence (*P* < 5 × 10^−5^) were excluded. We identified five SNPs, rs34143286, rs2465604, rs174448, rs2555277, and rs9564082, as LD-independent IVs (after the clumping process). The variance of oleic acid explained by genetic instruments was 29.1%. In our study, F statistics were all significantly >10, suggesting that our results were highly trustworthy and largely unaffected by weak IVs (Table 2).

**Table 2:** Detailed information on the SNPs associated with oleic acid and fatty acid (18:1)-OH.

**Exposure**	**SNP**	**EA/OA**	**EAF**	***beta***	***se***	**P**	**F**
Oleic acid	rs9564082	A/G	0.213	−0.109	0.027	4.246 × 10^−5^	33.005
	rs174448	A/G	0.643	−0.140	0.019	3.026 × 10^−13^	74.938
	rs2555277	A/G	0.378	0.083	0.019	9.349 × 10^−6^	26.822
	rs34143286	T/C	0.018	1.009	0.237	1.996 × 10^−5^	308.125
	rs2465604	T/C	0.821	−0.106	0.025	1.84 × 10^−5^	27.346
Fatty acid (18:1)-OH	rs1366632	G/T	0.424	−0.037	0.009	1.6 × 10^−5^	13.031
	rs72907101	T/C	0.011	−0.175	0.040	1.2 × 10^−5^	12.985
	rs13208968	A/G	0.477	0.036	0.009	2 × 10^−5^	12.601
	rs7941838	G/A	0.163	0.048	0.012	3.6 × 10^−5^	12.250
	rs9319312	G/T	0.141	−0.050	0.012	4.6 × 10^−5^	11.801
	rs12885354	G/A	0.139	0.052	0.012	3.1 × 10^−5^	12.612
	rs79758221	G/T	0.028	−0.115	0.026	8.2 × 10^−6^	14.029
	rs7274662	G/A	0.217	−0.047	0.010	4.3 × 10^−6^	14.629
	rs6005264	G/T	0.256	−0.046	0.010	2.5 × 10^−6^	15.710

EA/OA, Effect allele/Other allele; EAF, Effect allele frequency.

#### 3.1.2. Fatty acid (18:1)-OH

We selected nine independent SNP loci as IVs, namely rs72907101, rs1366632, rs79758221, rs7274662, rs6005264, rs7941838, rs12885354, rs9319312, and rs13208968. The explained variance of fatty acid (18:1)-OH was 59.8%. F statistics for each instrument–exposure association were all greater than the empirical threshold of 10, demonstrating a small likelihood of weak IVs (Table 2).

### 3.2. MR analysis for potential causal effects of oleic acid and fatty acid (18:1)-OH on DCM

In our study, oleic acid correlated positively with DCM risk. Based on the results obtained from the IVW approach, oleic acid has a suggestive causal association with increased risk of DCM, with a variance measured by IVs of 29.1%. (OR = 1.291, 95%CI: 1.044–1.595). The maximum likelihood approach confirmed this finding (OR = 1.296, 95%CI: 1.0149–1.595). However, the results of MR-Egger regression (OR = 1.249, 95%CI: 0.961–1.622) and the WME approach (OR = 1.271, 95%CI: 0.974–1.659) showed no statistical significance.

The IVW results for fatty acid (18:1)-OH showed a suggestive negative correlation with DCM risk, with a variance measured by IVs of 59.8% (OR = 0.402, 95%CI: 0.167–0.966). This negative correlation was proved by the maximum likelihood method (OR = 0.409, 95%CI: 0.168–0.997) and MRPRESSO method (OR = 0.402, 95%CI: 0.204–0.794). However, the results of MR-Egger regression (OR = 0.260, 95%CI: 0.052–1.300) and the WME method (OR = 0.324, 95%CI: 0.099–1.061) showed no statistical significance.

Ultimately, we employed the MR-Steiger test to determine the direction of the causal effect, confirming that the instrumentation of oleic acid and fatty acid (18:1)-OH both influenced susceptibility to DCM (*P* < 0.001), rather than vice versa, and that the orientation of the effect was fairly robust (Table 3).

**Table 3:** The results of the MR Steiger test.

**Exposure**	**Outcome**	***R*^2^ for exposure**	***R*^2^ for outcome**	***Correct_causal_direction***	***P_*steige*_***
Oleic acid	Dilated cardiomyopathy	0.015	7.34 × 10^−6^	TRUE	6.95 × 10^−28^
Fatty acid (18:1)-OH	Dilated cardiomyopathy	0.019	3.39 × 10^−5^	TRUE	1.10 × 10^−74^

*R*^2^, the variance of phenotype explained by SNPs.

### 3.3. Heterogeneity and pleiotropic evaluates

**Figure 3:**
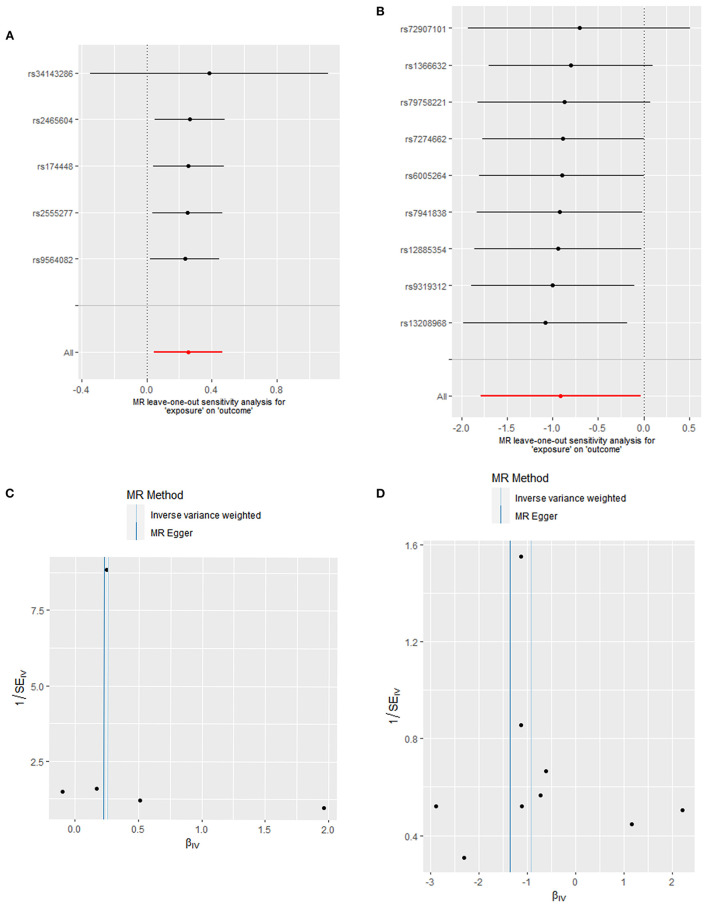
Heterogeneity and pleiotropic evaluates. Sensitivity analysis for SNP of **(A)** oleic acid and **(B)** fatty acid (18:1)-OH by “leave-one-out” method (Each black dot represents the odds ratio (OR) of the risk of developing DCM caused by increased oleic acid or fatty acid levels after excluding a specific SNP, red dots represent IVW estimates using all SNP, and horizontal segments represent 95% confidence intervals of the estimated values). Funnel plot to visualize the causal effect of **(C)** oleic acid and **(D)** fatty acid (18:1)-OH on DCM.

The results of Cochran's Q test and the MR-Egger test in the analysis for oleic acid and DCM proved no heterogeneity (*P* = 0.535) and no sensitive pleiotropy (*P* = 0.702), respectively. Further, there were no strange outliers in the included IVs' loci, which was mutually confirmed with the IVW results. Additionally, the results of the IVW random effect model and fixed effect model were consistent (OR: 1.290, 95%CI: 1.044–1.595, *P* = 0.018). After the calculations made using the “leave-one-out” method, we found that the included IVs affected these results indistinctively, suggesting our analysis was credible (Figure 3A). Additionally, funnel plots also confirmed that the causality between oleic acid and DCM was practically not affected by potential bias (Figure 3C).

Cochran's Q test and the horizontal pleiotropy of fatty acid (18:1)-OH suggested there was an absence of heterogeneity (*P* = 0.776) and gene pleiotropy (*P* = 0.548). The MRPRESSO test revealed no significant outliers. The “leave-one-out” sensitivity analysis affirmed the previous results (Figure 3B). The asymmetrical distribution of SNPs' loci in funnel plots also confirmed the absence of potential bias (Figure 3D).

## 4. Discussion

In this study, we used summary statistics from the GWAS database of large-scale clinical trials to execute a two-sample MR approach to assess the causal association between each available FA and DCM. Overall, we identified two FAs that have causal effects on DCM, oleic acid and fatty acid (18:1)-OH. Our results found that oleic acid may have an increased risk of DCM, while fatty acid (18:1)-OH may decrease the risk of DCM contrarily. The causal effects of other FAs on DCM, however, were found to be non-significant.

Oleic acid (C18:1 ω-9), a non-essential FA, is the main component of biofilm and also the highest content of monounsaturated fatty acid (MUFAs) provided by diets (~90% of all MUFAs) (34). Several authors have reported that oleic acid can significantly reduce nitric oxide levels in endothelial cells, which may be related to fatty acid-induced overproduction of superoxide and ROS in the mitochondrial electron transport chain (35). LURIC study for different types of omega-9 MUFAs showed that oleic acid was positively correlated with indicators of inflammation, vascular endothelial cell activation, heart failure, and even an increased risk of all-cause death (HR = 1.080, 95%CI:1.010–1.160) (36). Such risk effects of oleic acid on DCM may be related to its physiological toxicity for human beta cells (37). However, controversy still exists regarding this theory. In the PREDIMED trial conducted by Guasch-Ferré et al. MUFA intake was found to have a significant correlation with total cardiovascular disease (CVD) risk as a protective factor (HR = 0.630, 95%CI: 0.430–0.940) (38). This protection may be mediated by the synthesis of the endogenous signaling mediator NO_2_-OA (17) and hydroxylation to hydroxyl fatty acids in response to metabolic disturbances and increased inflammatory stress in the DCM (17). In general, these positive correlation results are consistent with ours, suggesting the causal effect of oleic acid on DCM.

Fatty acid (18:1)-OH is a monounsaturated hydroxyl FA (HFA) that contains 18 carbons. Few studies on HFAs have been reported, and the specific regulatory mechanism is currently unclear. Of these studies, 10-hydroxy-2-decenoic acid, the main FA of royal jelly, has been noted to inhibit endothelial vascular cell growth by inhibiting cell proliferation and migration (39) and to resist inflammation (40). Our findings propose that fatty acids (18:1)-OH, as a protective factor of DCM, has a significant causal effect on DCM risk. In a previous study, the corresponding HFAs were synthesized by hydroxylation with FAs as the substrate and nicotinamide adenine dinucleotide phosphate as the electron donor under the catalysis of cytochrome P450 (41). Based on the current evidence for HFAs, we speculate that this protective effect may be related to fatty acid hydroxyl fatty acid esters (FAHFAs). FAHFAs are a recently discovered group of endogenous lipid molecules that were first reported in *Cell* in 2014 (42). They are produced by the esterification of C16 and C18 FAs, such as oleic acid, palmitic acid and their corresponding HFAs (42). In an obese mouse model, FAHAFs have been found to inhibit the secretion of pro-inflammatory factors such as TNF and IL-1β (43). Research by the Beth Israel Deaconess Medical Center and Harvard Medical School has noted that FAHAFs can inhibit the endoplasmic reticulum stress response by reducing the activation of the JNK/MAPK pathway (42).Hence, FAHAFs are a promising class of anti-inflammatory lipids. To summarize, the protective effect of fatty acids (18:1)-OH on DCM may be related to their anti-inflammatory effects, which work directly or indirectly through esterification to form FAHAFs. Further experimental *in vivo* and clinical studies are needed to investigate this theory.

Different FAs have different effects on the risk of CVD. Most PUFAs are essential FAs that cannot be synthesized by the human body. Fish oil, which is rich in N-3 fatty acids, has been reported to potentially protect against cardiovascular disease by raising the threshold for arrhythmia, lowering blood pressure, and improving arterial and endothelial function (44). However, results from another randomized controlled trial suggest that fish oil supplementation may not reduce the risk of ventricular fibrillation and tachycardia, but may even trigger arrhythmias in some patients (HR = 1.76, 95%CI: 1.15–2.28). (19) Linoleic acid, an omega-6 PUFA, is the precursor of arachidonic acid, and can be converted into prostaglandins in the inflammatory cascade; it has been found to have a pro-inflammatory effect (45). Cells involved in inflammation usually have higher levels of omega-6 FAs and arachidonic acid. It is, however, interesting to note that linoleic acid and other omega-6 FAs may be risk factors for promoting inflammation or thrombosis (45), although a prospective clinical cohort study in *Circulation* has shown the cardioprotective effects of omega-6 PUFA intake (RR = 0.850, 95%CI: 0.780–0.920) (46). These conflicting results may be in part due to the different lineages, ages, and genders of people, sources of FAs and inclusion criteria used for different study groups. Our findings showed that the other 52 available FAs had no significant causal correlations with DCM. More studies of the mechanisms involved in the regulation of DCM by fatty acids are needed to verify whether the fatty acids themselves play a role or their metabolites do.

This is the first study to estimate the causal relationship between FAs and DCM. We explained the etiology of this relationship using the MR method and excluded the influence of confounding factors and reverse causal correlation to ensure the results were more reliable. At the same time, we conducted IVW, MR-Egger, MWE, maximum likelihood, MRPRESSO, and other calculation methods to validate the casual effect. In addition, genetic data of FAs and DCM were extracted from GWAS datasets, which aggregate a mass of clinical samples. However, our research has some limitations. First of all, most of the genetic data used were from Europeans, but some came from Asians. Although our data suggest that there may not be heterogeneity between these genetic variants, a small proportion of Asians may still lead to stratification among different ethnic groups. This issue requires further isolation of the raw data for validation. Secondly, the size of genetic effects could not be directly transformed into a clinical intervention effect. Considering that genetic variation is longer lasting, the causal effect estimated by MR is greater than the effect of clinical intervention generally. Thirdly, the non-linear relationship between exposure factors and outcomes could not be evaluated by the MR method. Fourthly, we cannot completely exclude possible diet-gene or gene-environment interactions that could have had an impact on our results. Furthermore, like other MR studies, we are unable to fully address the unobserved pleiotropies. It should be acknowledged that estimates of IVW effects are prone to bias when some instrumental SNPs exhibit horizontal pleiotropy (e.g., when FAs are measured by different measurements).

## 5. Conclusion and perspective

For the first time, we systematically analyzed the potential causal effect of 54 FAs on DCM. We identified a potential risk factor (oleic acid) and a potential protective factor [fatty acid (18:1)-OH] that were found to have suggestive significant causal effects on DCM events. On the one hand, more basic and clinical studies are needed to validate our results. On the other hand, the potential protective mechanism of fatty acid (18:1)-OH in DCM could be explored by detecting variations in oleic acid and the corresponding HFA and FAHFA, so as to provide theoretical references for the clinical prevention and treatment of DCM.

## Data availability statement

The original contributions presented in the study are included in the article/Supplementary material, further inquiries can be directed to the corresponding authors.

## Author contributions

YH, XC, and HW conceived the study, performed manuscript revision, and took accountability for all aspects of the work. JZ performed the data interpretation, drafted and revised the manuscript. QL designed the methodology and did the software analysis. JH, WX, and PL were in charge of supervision and administration. All authors have read and agreed to the published version of the manuscript.
